# Facile Solution Process of VO_2_ Film with Mesh Morphology for Enhanced Thermochromic Performance

**DOI:** 10.3390/ma15124129

**Published:** 2022-06-10

**Authors:** Zhao Yu, Zhe Wang, Bin Li, Shouqin Tian, Gen Tang, Aimin Pang, Dawen Zeng, Gopinathan Sankar

**Affiliations:** 1 State Key Laboratory of Silicate Materials for Architectures, Wuhan University of Technology (WUT), No. 122, Luoshi Road, Wuhan 430070, China; 268704@whut.edu.cn (Z.Y.); 290952@whut.edu.cn (Z.W.); libin625@whut.edu.cn (B.L.); 2 Science and Technology on Aerospace Chemical Power Laboratory, Hubei Institute of Aerospace Chemotechnology, Xiangyang 441003, China; tanggen518@126.com; 3 State Key Laboratory of Materials Processing and Die & Mould Technology, Huazhong University of Science and Technology, No. 1037, Luoyu Road, Wuhan 430074, China; dwzeng@mail.hust.edu.cn; 4 Department of Chemistry, University College London, London WC1H0AJ, UK; g.sankar@ucl.ac.uk

**Keywords:** VO_2_-based composite film, solution process, solar modulation, visible transmittance

## Abstract

The fabrication and applications of VO_2_ film continue to be of considerable interest due to their good thermochromic performance for smart windows. However, low visible transmittance (*T*_lum_) and solar modulation efficiency (∆*T*_sol_) impede the application of VO_2_ film, and they are difficult to improve simultaneously. Here, a facile zinc solution process was employed to control the surface structure of dense VO_2_ film and the processed VO_2_ film showed enhanced visible transmittance and solar modulation efficiency, which were increased by 7.5% and 9.5%, respectively, compared with unprocessed VO_2_ film. This process facilitated the growth of layered basic zinc acetate (LBZA) nanosheets to form mesh morphology on the surface of VO_2_ film, where LBZA nanosheets enhance the visible transmittance as an anti-reflection film. The mesh morphology also strengthened the solar modulation efficiency with small caves between nanosheets by multiplying the times of reflection. By increasing the zinc concentration from 0.05 mol/L to 0.20 mol/L, there were more LBZA nanosheets on the surface of the VO_2_ film, leading to an increase in the solar/near-infrared modulation efficiency. Therefore, this work revealed the relationship between the solution process, surface structure, and optical properties, and thus can provide a new method to prepare VO_2_ composite film with desirable performance for applications in smart windows.

## 1. Introduction

Energy saving in buildings has attracted much attention since there is a strong drive to minimize the use of non-renewable fuels, specifically fossil fuels [1]. Among various ways to save energy, smart windows based on thermochromic material have been widely explored, which can control the transmittance of sunlight in response to ambient temperature, intelligently, to keep the indoor temperature suitable for human beings, thus greatly reducing building energy consumption [2,3,4,5,6,7]. VO_2_ is one of the best thermochromic materials and is promising for the reversible transition between rutile structure and monoclinic structure [8,9,10,11], accompanied by an abrupt change in transmittance of near-infrared (NIR) light at 68 °C, which is somewhat higher than room temperature [12]. Ideally, VO_2_ film allows NIR light to enter to make the room warm in cold winter while it also keeps NIR light from going through in hot summer to sustain the cool indoor temperature [13]. However, low solar modulation efficiency (∆*T*_sol_), poor luminous transmittance (*T*_lum_), and the high phase transition temperature (*T*_c_) at 68 °C obstruct the application of VO_2_ films in smart windows [14].

To overcome these issues, many methods, including doping, composites with other materials, changing film structures, and surface engineering were employed [15,16,17,18]. Surface engineering is supposed to be an efficient method to promote the optical properties of VO_2_ film. Liu et al. prepared a highly ordered honeycomb-like structure, which enhanced *T*_lum_ significantly [15], and similarly, Ning et al. prepared various nanostructures through the sol-gel method in pure VO_2_, which increased *T*_lum_ from 20% to 36% [16]. Gao et al. fabricated nanoporous VO_2_(M) films to improve *T*_lum_ [17], and Xie et al. introduced a similar strategy of introducing a periodic porous structure into VO_2_(M) film to block infrared rather than visible light, which showed excellent *T*_lum_ and ∆*T*_sol_ [18]. In this sense, the surface structure has an important effect on the thermochromic properties of VO_2_ films, indicating that surface engineering is a possible way to enhance *T*_lum_ and ∆*T*_sol_ of VO_2_ films.

To control the surface structure of VO_2_ film, various methods were employed. The addition of protective layers is always used. These layers include TiO_2_ [19,20], V_2_O_5_ [21], SiO_2_ [22], SiN_x_ [23], HfO_2_ [24], ZnO [25], and so on. There is some strain between these layers and the VO_2_ film, which will have an important effect on the band structure of VO_2_ and thus control the phase transition temperature [26,27,28,29,30,31,32,33]. However, the additional protective layer always leads to the deteriorated optical performance of VO_2_ film with reduced solar modulation efficiency. Recently, ascorbic acid was used to process the VO_2_ surface to enhance its antioxidation and anti-acid properties [34]. An acid solution was utilized to etch pristine VO_2_ film prepared with magnetron sputtering to produce a karst landforms-like structure so that the solar modulation efficiency and visible transmittance were improved simultaneously [35]. Unfortunately, the karst landforms-like structure was not controlled easily because VO_2_ reacted with acid rather easily. Thus, it is of great significance to develop a facile method for the surface engineering of VO_2_-based film.

Inspired by these above works, and different from acid etching, the zinc solution process was employed to control the surface structure of VO_2_ films in this work. Here, zinc acetate was used to easily produce an LBZA compound on the surface of VO_2_ film at a low-temperature solution and likely had important effects on thermochromic performance because many Zn compounds such as ZnO or ZnS can help VO_2_ film enhance its thermochromic properties [25,36]. At first, VO_2_ films were synthesized by the magnetron sputtering method and then processed in the zinc solution. The obtained film exhibited a mesh morphology on the surface, and enhanced *T*_lum_ and ∆*T*_sol_ compared with unprocessed films. The effects of the zinc concentration on surface structures and thermochromic properties of the obtained films were investigated in detail. In addition, the relationship between surface structures and the thermochromic performance was revealed to explain the enhancement in thermochromic properties of the films.

## 2. Experiment and Characterization

### 2.1. VO_2_ Film Preparation with Magnetron Sputtering

VO_2_ films were deposited by magnetron sputtering on quartz glass substrates (2 cm × 2 cm). A V (99.95%) target was used for deposition. We evacuated the chamber to 3.0 × 10^−3^ Pa, then introduced Ar (99.99%) into the chamber and fixed the gas flow at 200 sccm without O_2_ in the atmosphere. The duration of the sputtering process was 15 min with a sputtering power of 75 W, and the temperature of the substrate was 298 K. This kind of VO_2_ film usually shows a compact structure and uniform particle distribution on the substrate with low visible transmittance. VO_2_ films obtained by magnetron sputtering were processed in a Zn^2+^ solution to manufacture multilayer composite films.

### 2.2. Post-Synthetic Solution Processing of VO_2_ Films

First, 1.3172 g (0.06 mol) of zinc acetate dihydrate (Zn (CH_3_COO)_2_·H_2_O) was dissolved in 60 mL of methanol (CH_3_OH) in a conical flask (100 mL) under vigorous stirring at room temperature to form a zinc solution, then subjected to ultrasonic radiation for 10 min before adding 15 mL of distilled water to the above solution. The solution was transformed from a transparent state to a white suspension during the process. The VO_2_ film with the substrate was dipped into the solution and the flask was covered with plastic film and processed in a water bath at 60 °C for 6 h. Finally, the VO_2_ film was taken out and washed with distilled water before heating on a hot plate at 60 °C.

### 2.3. Characterization

X-ray diffraction (XRD, D8Advance, CuKα, *λ* = 0.154178 nm under an output power of 3 kW) was employed to determine the phase structure in the film. The surface morphology, particle size, and composition of the films were examined with a scanning electron microscope (SEM, JSM-5610LV, JEOL, Tokyo, Japan) equipped with energy dispersed X-ray spectra (EDX). The surface structure was also examined with a scanning probe microscope (AFM, Nanoscope, IV/Nanoscope IV, VEECO, New York, NY, USA). The optical properties of the films (300–2500 nm) were measured using an ultraviolet-visible-near infrared spectrophotometer (UV-Vis-NIR, UV-3600, Shimadzu, Kyoto, Japan) at 20 °C and 90 °C, respectively. The integrated *T*_lum_ (380–780 nm) and ∆*T*_sol_ (380–2500 nm) were obtained from the following equation:

(1) $$T_{\mathrm{lum}/\mathrm{sol}}=\int \varphi_{\mathrm{lum}/\mathrm{sol}}\;\left(\lambda \right)T\left(\lambda \right)d\lambda /\int \varphi_{\mathrm{lum}/\mathrm{sol}}\;\left(\lambda \right)d\lambda$$

Here, *T*(*λ*) is the transmittance at wavelength *λ* of the film, *φ*_lum_(*λ*) is the standard luminous efficiency function for the photopic vision of human eyes, and *φ*_sol_(*λ*) denotes the AM 1.5 solar irradiance spectrum. Moreover, Δ*T*_sol/NIR_ is obtained from the following equation:

(2) $$\Delta T_{\mathrm{sol}}=T_{\mathrm{sol}}\left(20\;{}^{\circ}C\right)-T_{\mathrm{sol}}\left(90\;{}^{\circ}C\right)$$

## 3. Results and Discussion

### 3.1. Structure and Thermochromic Performance of Solution-Processed VO_2_-Based Film

**Figure 1:**
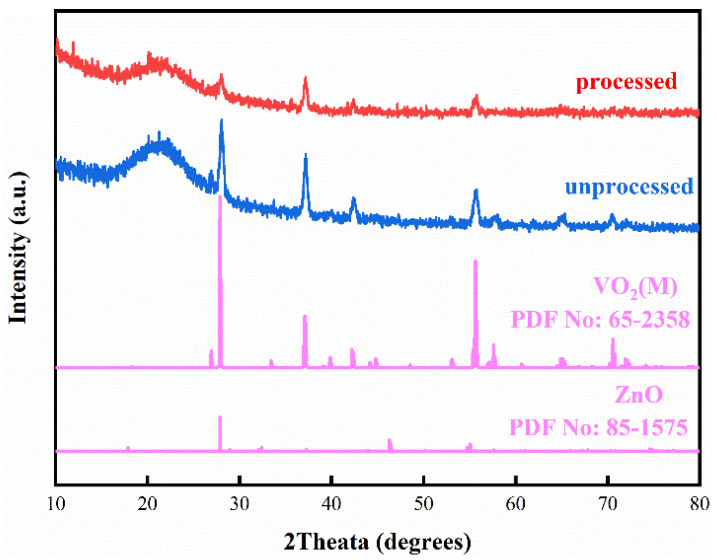
XRD patterns of obtained VO_2_ film after processing in Zinc solution.

VO_2_ film manufactured by magnetron sputtering was processed in a 0.10 mol/L zinc solution. Figure 1 presents the XRD patterns of processed and unprocessed samples. Three major diffraction peaks in the XRD patterns match VO_2_(M) (JPCDS No. 65-2358) well, suggesting no phase change in the sample. However, XRD failed to detect any phase related to zinc-containing compounds, possibly due to the poor crystallinity or small contents.

**Figure 2:**
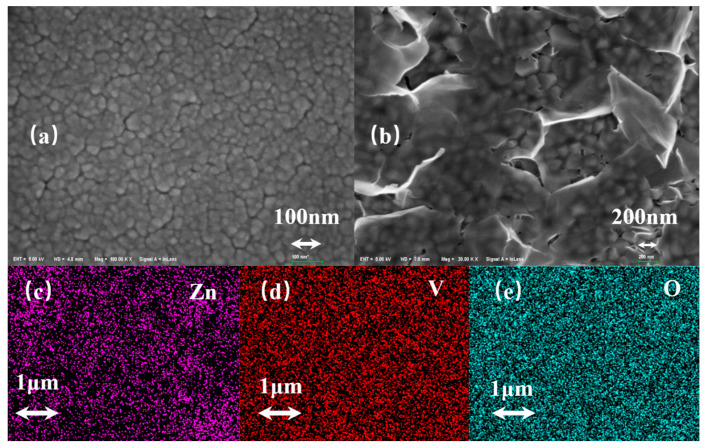
(**a**) SEM image of the film before (**a**) and after (**b**) solution process, and (**c**) Zn, (**d**) V, and (**e**) O element mapping of sample after solution process.

In order to characterize the surface structure of the VO_2_ film after the solution process, SEM and EDX were employed. Figure 2 shows the typical morphology of VO_2_ film before (a) and after (b) solution processing and the element mapping images of Zn (c), V (d), and O (e) on the film. In Figure 2b, the obtained film exhibits a mesh morphology, and the uniform nanoplates are distributed on the film surface, indicating that the nanoplates have grown on the film surface. Moreover, the three elements including Zn, V, and O are distributed uniformly on the film as shown in Figure 2c–e. The mesh morphology is similar to that of LBZA in the previous reports [37]. The related reaction for the formation of the LBZA nanostructure on the VO_2_ surface can be expressed as follows:

(3) $$\mathrm{Zn}{\left({\mathrm{CH}}_{3}\mathrm{COO}\right)}_{2}\cdot 2H_{2}O≜{\mathrm{Zn}}^{2+}+2{\mathrm{CH}}_{3}{\mathrm{COO}}^{-}+2H_{2}O$$

(4) $${\mathrm{Zn}}^{2+}+{\mathrm{CH}}_{3}{\mathrm{COO}}^{-}+H_{2}O≜\mathrm{LBZA}\;\left(\mathrm{layered}\;\mathrm{basic}\;\mathrm{zinc}\;\mathrm{acetate}\right)$$

**Figure 3:**
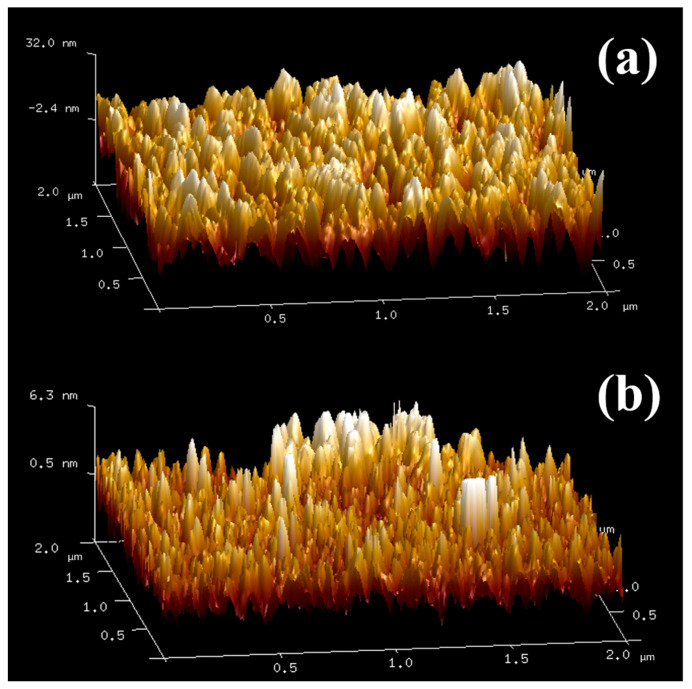
The morphology of VO_2_ unprocessed (**a**) and processed (**b**) films processed in zinc solution.

Figure 3 shows the roughness of the VO_2_ film before (a) and after (b) the solution process, which exhibits a great difference. Figure 3a shows the morphology of pure VO_2_ film prepared by magnetron sputtering and the film surface is nearly plane. In Figure 3b, the lower section of the film is supposed to be VO_2_ while the higher section has the typical morphology of nanoplates. The structures in the figure seem to be nanorods, but, in fact, they are nanoplates because the unit of height is of a nanometer scale while the width and length are of micrometer scale. As a result, nanoplates have grown on the VO_2_ film, which matches the above SEM results well. In addition, the solution-processed VO_2_ film exhibits a smaller height (6.3 nm) than that before the solution process (32 nm), likely because some VO_2_ grains in the film are dissolved in the solution [38].

**Figure 4:**
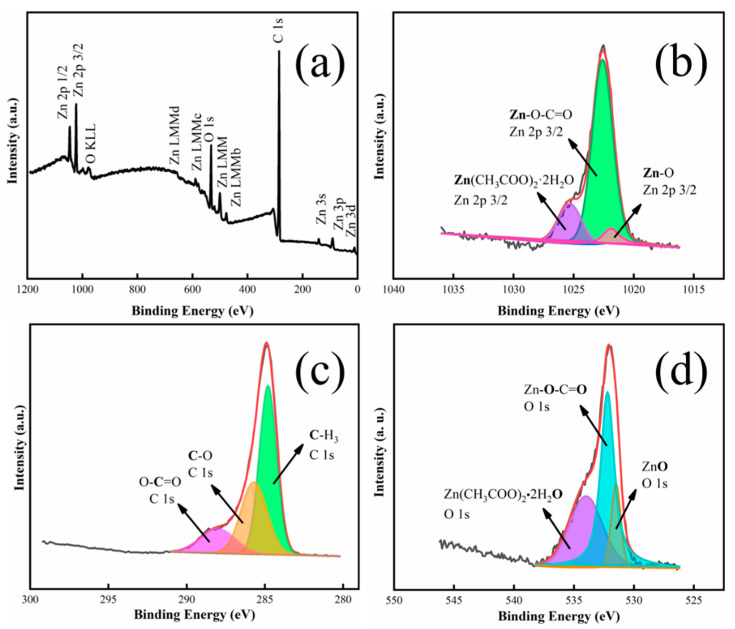
(**a**) XPS survey spectrum of a film processed in zinc solution and core-level spectra of (**b**) Zn 2p_3/2_, (**c**) C 1s, (**d**) O 1s.

Figure 4 shows the XPS spectra of the sample after the solution process. It can be seen that Zn, O, and C elements are present in the sample (Figure 4a). In Figure 4b, the XPS spectrum of Zn 2p_3/2_ can be divided into three peaks at 1021.9 eV, 1022.6 eV, and 1025.3 eV, respectively, indicating that the Zn element in the obtained sample exhibits three states in different chemical environments. The peak at 1021.9 eV matches crystalline ZnO well, whose binding energy value of Zn 2p_3/2_ is 1021.9 ± 0.1 eV [37], suggesting that the sample includes some ZnO. The peak at 1025.3 eV corresponds to Zn^2+^ in Zn(CH_3_COO)_2_·2H_2_O [39], indicating that Zn(CH_3_COO)_2_·2H_2_O likely exists in the sample. The peak at 1022.6 eV is assigned to the acetate zinc group [40], suggesting that there is LBZA with an acetate zinc group in the sample. It can be seen that the sample mainly includes LBZA, as well as a few ZnO and Zn(CH_3_COO)_2_·2H_2_O on its surface. This suggests the nanoplates on the film surface are mainly LBZA. In Figure 4c, the C 1s region includes three peaks detected to be 288.0 eV, 285.7 eV, and 284.9 eV, which correspond to O-C=O [41], C-O [42], and -CH_3_ [43]. In Figure 4d, the O 1s region can be also divided into three peaks at 534.0 eV, 532.0 eV, and 531.5 eV. The peak at 532.0 eV is ascribed to Zn-O-C=O [37] in LBZA while the 531.5 eV peak is ascribed to ZnO [44]. The 534.0 eV peak is related to O in the H_2_O of Zn(CH_3_COO)_2_·2H_2_O [45]. However, XPS failed to detect a V signal because the VO_2_ film is covered by LBZA and the detection depth of XPS is limited.

**Figure 5:**
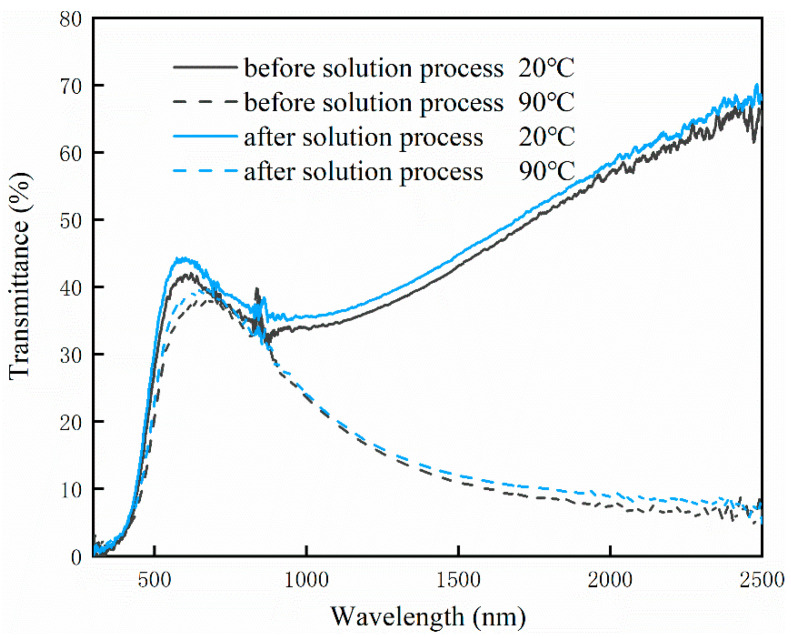
Solar transmittance spectra of VO_2_ films before and after the solution process.

Figure 5 exhibits the solar transmittance spectra of VO_2_ film before and after the solution process. At 20 °C, the transmittance of processed VO_2_ film shows notable promotion, especially in the short-wavelength area. After calculation, visible transmittance of the processed film at 20 °C is 40.1%, while unprocessed VO_2_ film is 37.3%. In addition, solar modulation efficiency is also promoted from 9.5% to 10.4%. This is possibly ascribed to the LBZA nanoplates on the surface of VO_2_ films. To investigate the enhancement in the thermochromic performance of solution-processed VO_2_-based film, the zinc concentration will be investigated as an important factor later.

### 3.2. The Effect of Zinc Concentration on Structure and Thermochromic Performance

**Figure 6:**
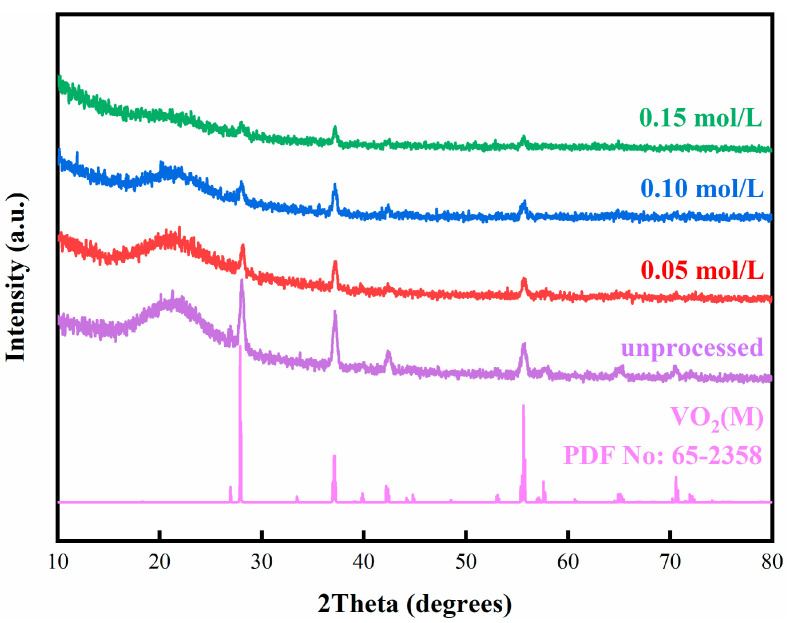
XRD patterns of obtained samples after processing in Zinc solution with different concentrations and standard VO_2_(M).

To explore the effects of zinc concentrations on the thermochromic properties of VO_2_ film, samples were processed in four different zinc concentrations (0.05 mol/L, 0.10 mol/L, 0.15 mol/L, and 0.20 mol/L) for 6 h, respectively. Figure 6 presents the XRD patterns of the obtained films. The green, blue, and red lines denote the films obtained at zinc concentrations of 0.15 mol/L, 0.10 mol/L, and 0.05 mol/L, respectively. All the diffraction peaks show great agreement with VO_2_(M) (JPCDS No. 65-2358), and no other peaks indexed to other compounds, indicating that obtained films are M-phase VO_2_ and the solution process does not change the phase structure of VO_2_ film obtained by magnetron sputtering. By increasing zinc concentration, the peak intensity decreased and the noncrystalline peak becomes weaker, which is attributed to the film thickness.

**Figure 7:**
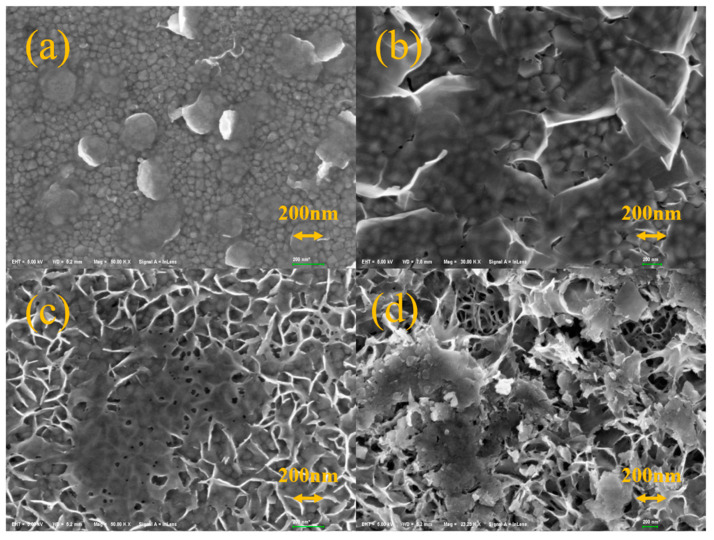
The SEM images of VO_2_ films after being processed in zinc solution with different concentrations: (**a**) 0.05 mol/L, (**b**) 0.10 mol/L, (**c**) 0.15 mol/L, (**d**) 0.20 mol/L.

**Figure 8:**
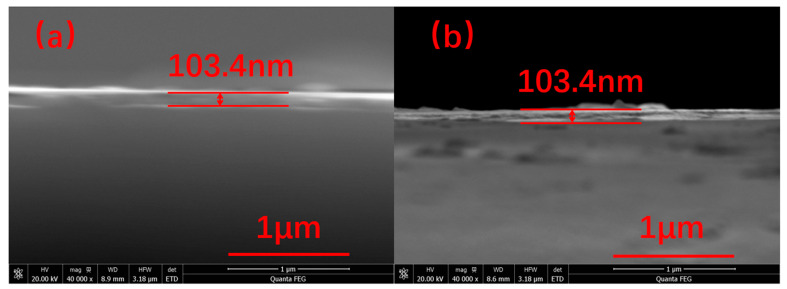
The cross-section SEM images of VO_2_ films before (**a**) and after (**b**) processed in 0.15 mol/L zinc solution.

**Figure 9:**
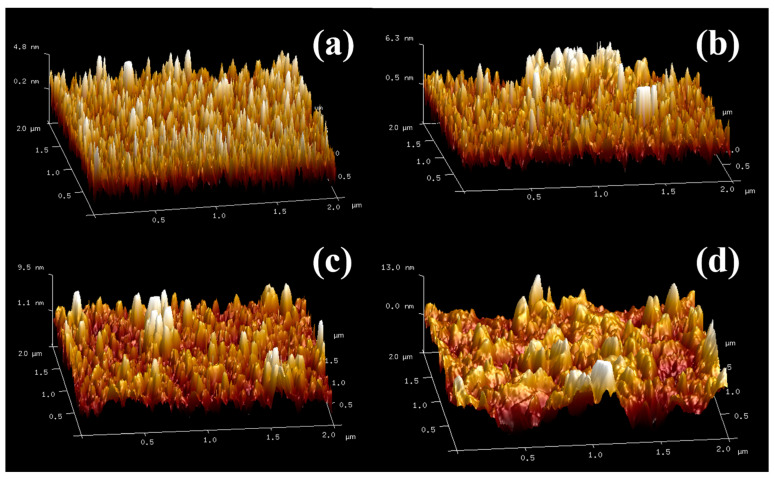
The AFM images for surface roughness of VO_2_ films after being processed in zinc solution with different concentrations: (**a**) 0.05 mol/L, (**b**) 0.10 mol/L, (**c**) 0.15 mol/L, (**d**) 0.20 mol/L.

The morphology of the obtained films was investigated further by SEM and AFM characterizations and the results are schematically illustrated in Figure 7, Figure 8 and Figure 9. Figure 7a shows the morphology of the film by zinc solution process at 0.05 mol/L. The nanoplates are thought to be layered basic zinc acetates, and the little grains below are the typical morphology of VO_2_. The structure corresponds well with Figure 9a. Although Figure 9a appears to display nanorods, they are, in fact, nanoplates because the unit of height is of nanometer scale while the width and length are of micrometer scale. At 0.05 mol/L, the nanoplates exhibit a scattered distribution on the surface. In Figure 7b–d, the nanoplates increase and finally cover the surface to form a mesh-like surface structure with an increasing zinc concentration from 0.10 to 0.20 mol/L. This is in good agreement with the AFM results in Figure 3 that the roughness of film increases with the increase in the zinc concentration. The film thickness was also characterized by SEM and the result is shown in Figure 8. It can be seen that the thickness of the VO_2_ film before the solution process was roughly 103.4 nm as shown in Figure 8a. After the solution process, the film thickness was also 103.4 nm. This indicates that the film thickness was not decreased.

**Figure 10:**
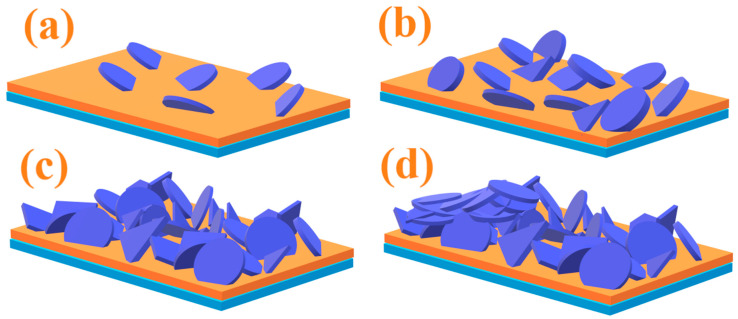
A demonstration of the structure change on the surface of the film after zinc solution process at 0.05 mol/L (**a**), 0.10 mol/L (**b**), 0.15 mol/L (**c**), 0.20 mol/L (**d**).

Based on the above results, the structure change of the film surface is clearly shown in Figure 10. The orange layer represents the VO_2_ film while the blue layer below represents the glass substrate. The violet plates above represent the nanoplates of LBZA, which multiply with the increasing zinc concentration.

**Figure 11:**
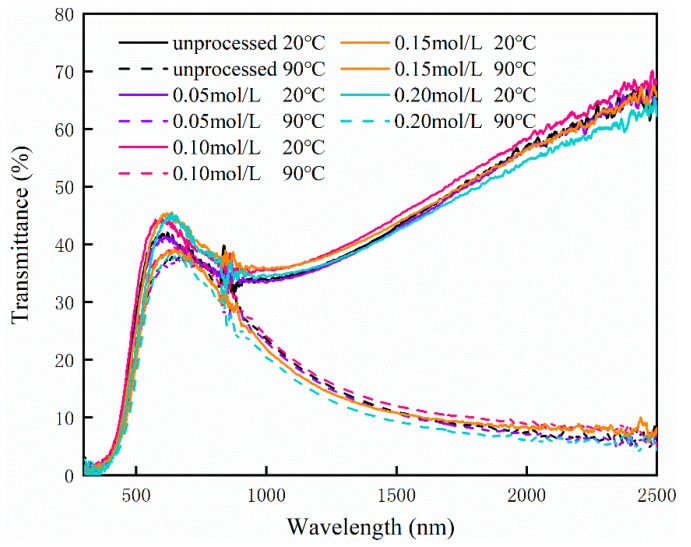
Solar transmittance spectra of VO_2_ films processed in zinc solution with 0.05 mol/L, 0.10 mol/L, 0.15 mol/L, and 0.20 mol/L.

To characterize the thermochromic properties of the obtained samples after the solution process, their solar transmittance (300–2500 nm) was measured with a UV-vis-NIR spectrophotometer at 20 °C as well as 90 °C, and the transmittance is shown in Figure 11. These four curves are almost the same, indicating that zinc concentration has no obvious effect on the solar transmittance of the obtained films. From the data in Figure 11, the visible transmittance, solar modulation efficiency, and near-infrared modulation efficiency were calculated and are exhibited in Table 1. With the increase in the zinc concentration in the solution, the visible transmittance increased at first and then decreased. When the zinc concentration is 0.10 mol/L, the visible transmittance of the VO_2_ film reached the maximum value (40.1%). As the zinc concentration increased, the solar modulation efficiency and infrared modulation efficiency also increased. When the zinc concentration was 0.20 mol/L, the solar/NIR modulation efficiency reached the maximum. However, the difference decreases when the zinc concentration is larger than 0.15 mol/L, so this can be ignored. It is obvious that, at lower zinc concentrations, the transmittance from 300 nm to 2500 nm increased at both 20 °C and 90 °C, which causes higher visible transmittance. When the zinc concentration in the solution increases, the transmittance from 300 nm to 2500 nm at 20 °C remains high while the transmittance from 300 nm to 2500 nm at 90 °C decreases, leading to an enhanced solar modulation efficiency. Specifically, when the zinc concentration is 0.15 mol/L, the film shows a visible transmittance of 38.2% at 20 °C, a solar modulation efficiency of 11.3%, and an infrared modulation efficiency of 20.3%, which is better than that of most VO_2_ films in previous works [15,16,17,46] as shown in Table 2.

**Figure 12:**
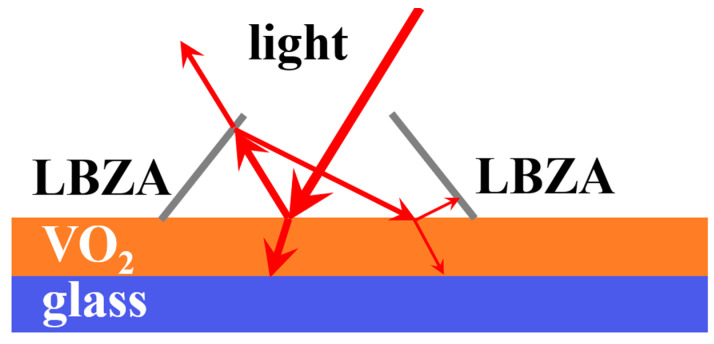
The principle of reflection and transmission on LBZA/VO_2_ film.

The enhancement in visible transmittance and solar modulation is related to the nanostructure of LBZA. There are many small cavities on the surface of LBZA nanoplates in Figure 12. Light reflected by VO_2_ is more likely to be reflected by LBZA again. After the second reflection, some light is reflected onto VO_2_ again, and the third reflection on VO_2_ will also occur in the same way. Compared with pure VO_2_ film, the VO_2_/LBZA film can reflect the light more times, and the reflected light had a second chance to go through the film. In this sense, more light can transmit through the composite film, leading to an increased visible transmittance, which is in good agreement with the results in Figure 5. With the occurrences of light reflection increased, more near-infrared light is absorbed by the VO_2_ film, resulting in an increase in the solar modulation efficiency. It seems that some light is trapped in the small cavities and continues reflection until escaping, leading to an enhanced solar modulation efficiency. Here, we assume the reflectivity of LBZA is R, then the light reflected by LBZA back to VO_2_ is R ∗(1−T), in which ΔT=R ∗(1−T)∗T transmits the film with a second chance. Since R is constant, the additional transmittance depends on T (transmittance of VO_2_). Obviously, the additional transmittance obtains the maximum with T at 50%. As shown in Figure 5, the transmittance at 20 °C is always closer to 50% than that at 90 °C, so the increase is always larger at 20 °C. Since ∆*T*_sol_ = *T*_sol_(20 °C) − *T*_sol_(90 °C) and *T*_sol_(20 °C) increases more, ∆*T*_sol_ also increases at the same time. As a result, the enhancement in both *T*_lum_ and ∆*T*_sol_ can be attributed to the nanostructure of LBZA on the surface of the VO_2_ film. In addition, the thermochromic performances of the obtained VO_2_ composite film were similar after 100 thermal cycles, leading to good stability. This is likely because the LBZA/VO_2_ composite film is very stable at a temperature below 100 °C.

## 4. Conclusions

In this work, the zinc solution process was used to prepare LBZA nanoplates on the surface of VO_2_ film. The obtained VO_2_ film exhibited enhanced thermochromic performance compared with unprocessed VO_2_ film: The visible transmittance was increased from 37.3% up to 40.1%, solar modulation efficiency was promoted from 9.5% to 10.4%, and the infrared modulation efficiency was improved from 17.4% to 18.8%. The enhancement was likely attributed to the formed LBZA nanoplates, which can increase the occurrences of solar light reflection. In addition, by increasing the zinc concentration, the visible transmittance was increased at first and then decreased, while the solar modulation efficiency and infrared modulation efficiency were increased. Therefore, zinc solution processing is a facile method to control the surface structure of VO_2_ film and thus has important effects on the thermochromic performance, which can shed light on the preparation of VO_2_ films with high performance for smart windows.

## Figures and Tables

**Table 1:** Thermochromic properties of different films processed in solutions with different zinc concentrations.

Sample	Zinc Concentration (mol/L)	*T*_lum_ (20 °C)	*T*_lum_ (90 °C)	∆*T*_sol_	∆*T*_NIR_
a	-	37.3%	31.2%	9.5%	17.4%
b	0.05	36.2%	30.6%	9.8%	18.4%
c	0.10	40.1%	33.5%	10.4%	18.8%
d	0.15	38.2%	32.1%	11.3%	20.3%
e	0.20	35.5%	30.1%	11.3%	20.5%

**Table 2:** Comparison of thermochromic performance of this work with previous works.

Samples	Thermochromic Properties		References
	*T*_lum_ (%)	∆*T*_sol_ (%)	
VO_2_ with honeycomb structure	95.4%	5.5%	Liu et al. [15]
VO_2_ with nanoporous structure	33.1%	2.2%	Wang et al. [16]
VO_2_ with nanoporous structure	43.4%	14.1%	Kang et al. [17]
VO_2_ with periodic porous	67.5%	7.5%	Zhou et al. [18]
AZO/PDLC/VO_2_	39.9%	9.95%	Sang et al. [46]
LBZA/VO_2_	40.1%	10.4%	This work (sample c)
LBZA/VO_2_	38.2%	11.3%	This work (sample d)

## References

[1] Kamalisarvestani M., Saidur R., Mekhilef S., Javadi F. (2013). Performance, materials and coating technologies of thermochromic thin films on smart windows. Renew. Sustain. Energy Rev..

[2] Granqvist C.G. (2007). Transparent conductors as solar energy materials: A panoramic review. Sol. Energy Mater. Sol. Cells.

[3] Kanu S.S., Binions R. (2009). Thin films for solar control applications. Proc. R. Soc. A Math. Phys. Eng. Sci..

[4] Li C., O’Halloran K.P., Ma H., Shi S. (2009). Multifunctional multilayer films containing polyoxomctalatcs and bismuch oxide nanoparticles. J. Phys. Chem. B.

[5] Baetens R., Jelle B.P., Gustavsen A. (2010). Properties, requirements and possibilities of smart windows for dynamic daylight and solar energy control in buildings: A state-of-the-art review. Sol. Energy Mater. Sol. Cells.

[6] Granqvist C.G. (2006). Electrochromic materials: Out of a niche. Nat. Mater..

[7] Glaster H.J. (2008). History of the development and industrial production of low thermal emissity coatings for high heat insulating glass units. Appl. Opt..

[8] Chen S., Ma H., Yi X., Xiong T., Wang H., Ke C. (2004). Smart VO2 thin film for protection of sensitive infrared detectors from strong laser radiation. Sens. Actuators A Phys..

[9] Smith A.W. (1973). Optical storage in VO_2_ films. Appl. Phys. Lett..

[10] Case F.C. (1991). Improved VO_2_ thin films for infrared switching. Appl. Opt..

[11] Bock D.C., Marschilok A.C., Takeuchi K.J., Takeuchi E.S. (2012). Batteries used to power implantable biomedical devices. Electrochimica Acta.

[12] Morin F.J. (1959). Oxides Which Show a Metal-to-Insulator Transition at the Neel Temperature. Phys. Rev. Lett..

[13] Warwick M.E.A., Binions R. (2013). Advances in thermochromic vanadium dioxide films. J. Mater. Chem. A.

[14] Wu C., Feng F., Xie Y. (2013). Design of vanadium oxide structures with controllable electrical properties for energy applications. Chem. Soc. Rev..

[15] Liu M., Su B., Kaneti Y.V., Chen Z., Tang Y., Yuan Y., Gao Y., Jiang L., Jiang X., Yu A. (2016). Dual-Phase Transformation: Spontaneous Self-Template Surface-Patterning Strategy for Ultra-transparent VO_2_ Solar Modulating Coatings. ACS Nano.

[16] NNing W., Yizhong H., Magdassi S., Mandler D., Hai L., Yi L. (2013). Formation of VO_2_ zero-dimensional/nanoporous layers with large supercooling effects and enhanced thermochromic properties. RSC Adv..

[17] Kang L., Gao Y., Luo H., Chen Z., Du J., Zhang Z. (2011). Nanoporous thermochromic VO_2_ films with low optical constants, enhanced luminous transmittance and thermochromic properties. ACS Appl. Mater. Inter..

[18] Zhou M., Bao J., Tao M., Zhu R., Lin Y., Zhang X., Xie Y. (2013). Periodic porous thermochromic VO_2_(M) films with enhanced visible transmittance. Chem. Commun..

[19] Sun G., Cao X., Li X., Bao S., Li N., Liang M., Gloter A., Gu H., Jin P. (2016). Low-temperature deposition of VO2 films with high crystalline degree by embedding multilayered structure. Sol. Energy Mater. Sol. Cells.

[20] Miller M.J., Wang J.L. (2016). Multilayer ITO/VO_2_/TiO_2_ thin films for control of solar and thermal spectra. Sol. Energy Mater. Sol. Cells.

[21] Liu H., Wan D., Ishaq A., Chen L., Guo B., Shi S., Luo H., Gao Y. (2016). Sputtering deposition of sandwich-structure V_2_O_5_/Metal/V_2_O_5_ multilayers for the preparation of high-performance thermally sensitive VO_2_ thin films with selectivity of VO_2_ (B) and VO_2_ (M) polymorph. ACS Appl. Mater. Inter..

[22] Kang J., Liu J., Shi F., Dong Y., Song X., Wang Z., Tian Z., Xu J., Ma J., Zhao X. (2022). Facile fabrication of VO_2_/SiO_2_ aerogel composite films with excellent thermochromic properties for smart windows. Appl. Surf. Sci..

[23] Long S., Cao X., Li N., Xin Y., Sun G., Chang T., Bao S., Jin P. (2018). Application-oriented VO_2_ thermochromic coatings with composite structures: Optimized optical performance and robust fatigue properties. Sol. Energy Mater. Sol. Cells.

[24] Zong H., Zhou D., Yan L., Li M., Qiao W., Zhang S., Hu Q., Bian L. (2022). Preparation and characterization of HfO_2_/VO_2_/HfO_2_ sandwich structures with low phase transition temperature, excellent thermochromic properties, and superior durability. Ceram. Int..

[25] Fang Z., Tian S., Li B., Liu Q., Liu B., Zhao X., Sankar G. (2021). VO_2_/ZnO bilayer films with enhanced thermochromic property and durability for smart windows. Appl. Surf. Sci..

[26] Goodenough J.B. (1971). The two components of the crystallographic transition in VO_2_. J. Solid State Chem..

[27] Zylbersztejn A.M.N.F., Mott N.F. (1975). Metal-insulator transion in vanadium dioxide. Phys. Rev. B.

[28] Gomez-Heredia C.L., Ramirez-Rincon J.A., Bhardwaj D., Rajasekar P., Tadeo I.J., Cervantes-Lopez J.L., Ordonez-Miranda J., Ares O., Umarji A.M., Drevillon J. (2019). Measurement of the hysteretic thermal properties of W-doped and undoped nanocrystalline powers of VO_2_. Sci Rep..

[29] Brown B.L., Lee M., Clem P.G., Nordquist C.D., Jordan T.S., Wolfley S.L., Leonhardt D., Edney C., Custer J.A. (2013). Electrical and optical characterization of the metal-insulator transition temperature inCr-doped VO_2_ thin films. J. Appl. Phys..

[30] Chen S., Wang Z., Ren H., Chen Y., Yan W., Wang C., Li B., Jiang J., Zou C. (2019). Gate-controlled VO_2_ phase transition for high-performance smart windows. Sci. Adv..

[31] Chen H.W., Li C.I., Ma C.H., Chu Y.H., Liu H.L. (2021). Strain engineering of optical properties in transparent VO_2_/muscovite heterstructures. Phys. Chem. Chem. Phys..

[32] D’Elia A., Grazioli C., Cossaro A., Li B.W., Zou C.W., Rezvani S.J., Pinto N., Marcelli A., Coreno M. (2021). Strain mediated filling control nature of the metal-insulator transition of VO_2_ and electron correlation effects in nannostructured films. Appl. Surf. Sci..

[33] Nazari M., Zhao Y., Kuryatkov V.V., Fan Z.Y., Bernussi A.A., Holtz M. (2013). Temperature dependence of the optical properties of VO_2_ deposited on sapphire with different orientations. Phys. Rev. B.

[34] Chen Y., Shao Z., Yang Y., Zhao S., Tao Y., Yao H., Luo H., Cao X., Jin P. (2019). Electrons-donating derived dual-resistant curst of VO_2_ nano-particles via ascorbic acid treament for highly stable smart windows appplications. ACS Appl. Mater. Inter..

[35] Long S., Cao X., Wang Y., Chang T., Li N., Jin L., Ma L., Xu F., Sun G., Jin P. (2020). Karst landform-like VO_2_ single layer solution: Controllable morphology and excellent optical performance for smart glazing applications. Sol. Energy Mater. Sol. Cells.

[36] Ji H., Liu D., Zhang C., Cheng H. (2018). VO_2_/ZnS core-shell nanoparticle for the adaptive infrared camouflage application with modified color and enhanced oxidation resistance. Sol. Energy Mater. Sol. Cells.

[37] Wang Z., Li B., Tian S., Liu B., Zhao X., Zhou X., Tang G., Pang A. (2021). Acid Solution Processed VO_2_-Based Composite Films with Enhanced Thermochromic Properties for Smart Windows. Materials.

[38] Liang M.-K., Limo M.J., Rabada A.S., Roe M.J., Perry C.C. (2014). New Insights into the Mechanism of ZnO Formation from Aqueous Solutions of Zinc Acetate and Zinc Nitrate. Chem. Mater..

[39] Mar L.G., Timbrell P.Y., Lamb R.N. (1993). An XPS study of zinc oxide thin film growth on copper using zinc acetate as a precursor. Thin Solid Films.

[40] Wagner C.D., Riggs W.M., Davis L.E., Moulder J.F., Muilenberg G.E. (1979). Handbook of X-ray Photoelectron Spectroscopy.

[41] Onyiriuka E. (1993). Zinc phosphate glass surfaces studied by XPS. J. Non-Crystalline Solids.

[42] Augustynski J., Koudelka M., Sanchez J., Conway B.E. (1984). ESCA study of the state of iridium and oxygen in electrochemically and thermally formed iridium oxide films. Electroanal. Chem..

[43] Clark D.T., Feast W.J., Tweedale P.J., Thomas H.R. (1980). ESCA applied to polymers. XXVI. Investigation of a series of aliphatic, aromatic, and fluorine-containing polycarbonates. J. Polym. Sci. Polym. Chem. Ed..

[44] Chen J.J., Jiang Z.C., Zhou Y., Chakraborty B.R., Winograd N. (1995). Spectroscopic studies of methanol deposition on Pd[lcd]111[rcub]. Surf. Sci..

[45] Clark D.T., Kilcast D., Musgrave W.K.R. (1971). Molecular core binding energies for some monosubstituted benzenes, as determined by X-ray photoelectron spectroscopy. J. Chem. Soc. D.

[46] Sang J., Zhu W., Feng Y., Liu Y., Shang J., Sun J., Guo L., Zhang Y., Zhao S., Chigrinov V. (2021). Smart Windows with a VO_2_ Thin Film as a Conductive Layer for Efficient and Independent Dual-Band Modulation. ACS Appl. Electron. Mater..

